# A Dichalcogenide-Protected Pt@Au_12_@Ag_8_ Nanocluster

**DOI:** 10.1021/acs.inorgchem.6c04087

**Published:** 2026-10-15

**Authors:** Tzu-Hao Chiu, Michael N. Pillay, Samia Kahlal, Jean-Yves Saillard, C. W. Liu

**Affiliations:** † Department of Chemistry, 63373 National Dong Hwa University, Hualien 97401, Taiwan (Republic of China); ‡ Univ Rennes, CNRS, ISCR-UMR 6226, F-35000 Rennes, France

## Abstract

Precise control over the location of heterometal atoms in alloy nanoclusters is essential for understanding how metal distribution governs superatomic structure and properties. Herein we show that PtAg_20_[S_2_P­(OPr)_2_]_12_ (**PtAg**_**20**_) can serve as a structurally defined precursor for constructing a PtAu_12_ superatom copassivated by ligands and Ag atoms through redox-mediated Au-for-Ag exchange. Treatment of **PtAg**_**20**_ with an excess of Au­(I) affords PtAu_12_Ag_8_[S_2_P­(OPr)_2_]_12_ (**PtAu**_**12**_ **Ag**_**8**_), in which substitution of Ag by Au occurs exclusively on the PtAg_20_ icosahedral core. Single-crystal X-ray diffraction reveals a compact PtAu_12_ core encapsulated by two Ag_4_(dtp)_6_ (dtp = dithiophosphate) units. Unlike all the known silver-rich isoelectronic nanoclusters featuring a centered icosahedron protected by a Ag_8_(dtp)_12_ outer shell that adopt *C*_3_-type or *C*_3_-derived shell arrangements, **PtAu**_**12**_ **Ag**_**8**_ displays a *C*_2_ geometry. Spectroscopic and mass-spectrometric measurements support the formation of the Au-rich cluster, while DFT calculations identify the [PtAu_12_]^4+^ core as an eight-electron superatom with a closed-shell 1S^2^1P^6^1D^0^ electronic configuration. This work demonstrates that selective replacement of the icosahedral cage can generate not only a new trimetallic superatomic core but also an unusual reconstruction of the surrounding Ag–ligand passivating layer.

## Graphical abstract

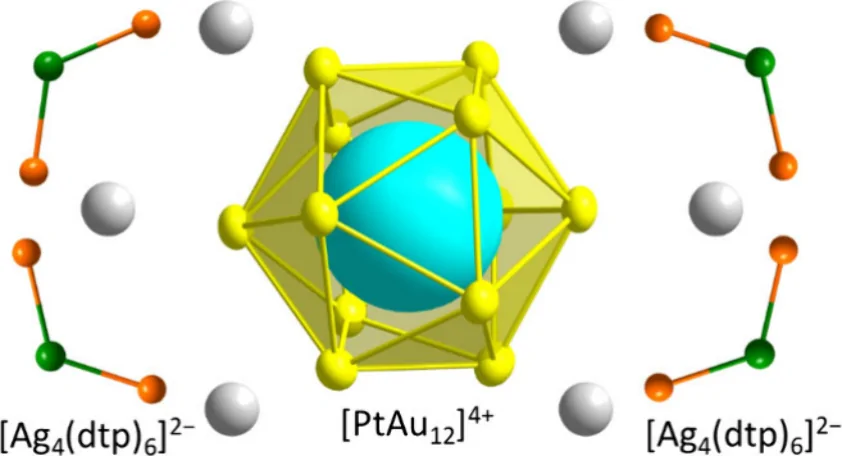

Ligand-protected metal nanoclusters have attracted considerable attention because their atomically precise structures and quantized electronic states provide opportunities for applications in catalysis, materials science, and biomedicine.^1–8^ Heterometal doping is one of the most effective strategies for diversifying their structures and tuning their properties.^8–10^ In particular, centered icosahedral superatoms represent an important class of ligand-protected nanoclusters, in which the electronic structure can often be described by a shell-closing superatomic model.

For ligand-protected eight-electron superatoms containing a centered M@M′_12_ icosahedral core, heterometal substitution can occur at several distinct sites: the central metal atom, the 12 metal atoms forming the icosahedral cage, and the metal atoms in the passivating layer. Substitution at the central site has been widely explored, with examples involving group 10 to group 8 metals.^11–31^ Replacement of the icosahedral cage atoms provides another powerful approach to modify the superatomic core directly. In Ag-rich superatoms, Au is an especially attractive substituent because its higher reduction potential can drive Ag-to-Au replacement through metal-exchange reactions.^13,32–39^ In contrast, substitution of metal atoms in the passivating layer mainly alters the surface coordination environment and can influence stability, reactivity, and catalytic performance without necessarily changing the electronic structure of the superatom.^39–42^

Despite the broad use of redox-driven Au-for-Ag exchange in Ag-rich nanoclusters, achieving site-selective Au incorporation remains challenging. In many alloyed Au/Ag systems, the incoming Au atoms are not necessarily devoted to substituting the Ag atoms of the superatomic core, because both core and peripheral Ag atoms may participate in the exchange.^32–39^ Consequently, it remains difficult to construct an atomically precise structure in which a [PtAu_12_]^4+^ icosahedral core is selectively protected by a Ag–ligand passivating layer. Furthermore, a fundamental understanding of how localized framework substitutions influence the overall geometric symmetry and topological rearrangement of the surrounding ligand shell is still largely unexplored.

Motivated by these challenges, our primary objective herein is to perform a regioselective galvanic exchange of the icosahedral [PtAg_12_] cage in parent PtAg_20_[S_2_P­(OPr)_2_]_12_ (**PtAg**_**20**_) to construct the trimetallic PtAu_12_Ag_8_[S_2_P­(OPr)_2_]_12_ (**PtAu**_**12**_ **Ag**_**8**_). Structural, spectroscopic, mass-spectrometric, and theoretical analyses reveal that the PtAg_12_ core is converted into a PtAu_12_ one, while the Ag_8_(dtp)_12_ (dtp = dithiophosphate) composition of the passivating homoleptic shell is retained but its topology is substantially modified. Thus, *complete Ag-to-Au replacement in the icosahedral cage is unexpectedly accompanied by reconstruction of the outer Ag–ligand passivating layer*.

**PtAu**_**12**_ **Ag**_**8**_ was synthesized through a redox-mediated metal-exchange reaction by treating **PtAg**_**20**_ with an excess amount of Au­(SMe_2_)­Cl. After completion of the reaction, purification with hexane and recrystallization afforded **PtAu**_**12**_ **Ag**_**8**_ as a pure product; all subsequent spectroscopic measurements were performed on isolated crystalline material. The ESI-MS spectrum shows two dominant signals assignable to [**PtAu**_**12**_ **Ag**_**8**_ + Ag]^+^ and [**PtAu**_**11**_ **Ag**_**9**_ + Ag]^+^ (Figure 1a). In addition, a weaker but distinguishable signal corresponding to [**PtAu**_**12**_ **Ag**_**8**_ + Au]^+^ was also observed, further supporting the formation of the **PtAu**_**12**_ **Ag**_**8**_ cluster. The distribution of peaks spaced by ∼89 Da originates from gas-phase metal exchange and fragmentation during electrospray ionization ([**PtAu_*x*_Ag_20–*x*_** + Ag]^+^, *x* = 8–12) (Table S4). The ^31^P NMR spectrum of **PtAg**_**20**_ shows one major resonance at 104.5 ppm at room temperature. After metal exchange, the ^31^P NMR spectrum of **PtAu**_**12**_ **Ag**_**8**_ displays two sets of resonances at 102.5 and 101.1 ppm (Figure 1b) with an integrated area ratio of 1:2. This 1:2 ratio reflects two distinct ligand coordination environments undergoing time-averaged dynamic coordination exchange in solution, consistent with the molecular symmetry observed in the solid state. The optical absorption spectrum also changes after Au incorporation. **PtAg**_**20**_ exhibits a relatively distinct absorption band at 412 nm, whereas **PtAu**_**12**_ **Ag**_**8**_ shows less resolved absorption features around 420, 520, and 700 nm (Figure 1c).

**Figure 1:**
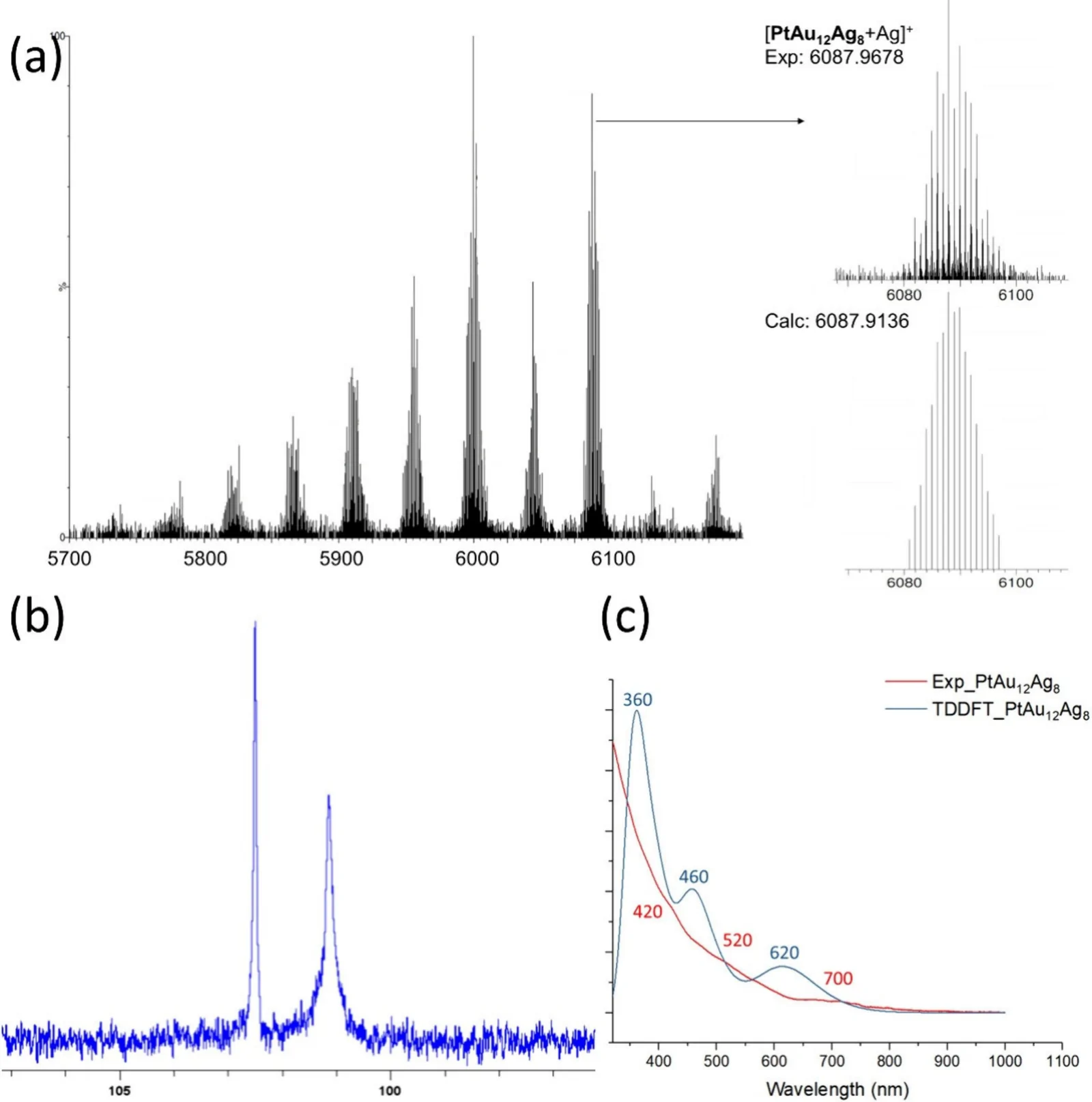
Characterization of **PtAu**_**12**_ **Ag**_**8**_. (a) ESI-MS spectrum of **PtAu**_**12**_ **Ag**_**8**_. Insets show the experimental (top) and simulated (bottom) isotopic distributions of the [**PtAu**_**12**_ **Ag**_**8**_ + Ag]^+^ ion peak. (b) ^31^P NMR spectrum of **PtAu**_**12**_ **Ag**_**8**_. (c) UV–vis absorption spectrum of **PtAu**_**12**_ **Ag**_**8**_.

**PtAu**_**12**_ **Ag**_**8**_ crystallizes in the monoclinic *P*2_1_/*n* space group. Its structure consists of a compact **PtAu**_**12**_ icosahedron protected by a passivating layer composed of eight Ag atoms and 12 [S_2_P­(OPr)_2_]^−^ ligands. The eight Ag atoms are equally divided into two sets, each capping adjacent triangular faces of the icosahedron, and each Ag atom is coordinated by three S atoms from three different ligands (Figure 2). The 12 P atoms of the ligands define a slightly distorted bicapped pentagonal prism, in which the torsion angle between the two pentagonal faces is 18° (Figure S1). Notably, each ligand binds to the PtAu_12_ core through only one S atom (Figure S2). The overall molecular structure exhibits *C*_2_ symmetry. In this framework, the 12 ligands are partitioned in a 4:8 ratio across the cluster: four symmetry-equivalent ligands occupy equatorial sites, corresponding to the sharp ^31^P resonance at 102.5 ppm, while the remaining eight ligands populate the two capping [Ag_4_(dtp)_6_]^2–^ units, where rapid intrashell fluxionality across labile capping positions accounts for the broadened resonance at 101.1 ppm. Compared with **PtAg**_**20**_, the passivating layer surrounding the superatomic core undergoes a pronounced rearrangement. We propose that this structural reconstruction arises from the preference of Au for a lower-coordination environment. In the parent **PtAg**_**20**_ structure, some of the icosahedral Ag atoms are connected to two S atoms,^12,23,43^ which would be less favorable for Au. Among all the known silver-rich isoelectronic nanoclusters composed of a centered icosahedral core passivated by a Ag_8_(dtp)_12_ outer layer, **PtAu**_**12**_ **Ag**_**8**_ represents the first example featuring a shell arrangement having *C*_2_ symmetry, leading to *C*_2_ for the whole **PtAu**_**12**_ **Ag**_**8**_ cluster. Previously reported examples either exhibit *C*_3_ symmetry or can be regarded as deriving from this *C*_3_ topology (Figure S3). Consistent with the slightly smaller atomic radius of Au compared with Ag, both the Pt–Au and Au–Au distances in **PtAu**_**12**_ **Ag**_**8**_ are slightly shorter than the corresponding Pt–Ag_ico_ and Ag_ico_–Ag_ico_ distances in **PtAg**_**20**_. The more compact nature of the PtAu_12_ icosahedron is also reflected in its smaller cage volume. The Au_12_ cage in **PtAu**_**12**_ **Ag**_**8**_ has a volume of 52.26 Å^3^, compared with 53.87 Å^3^ for the Ag_12_ cage in **PtAg**_**20**_ (Table 1).

**Figure 2:**
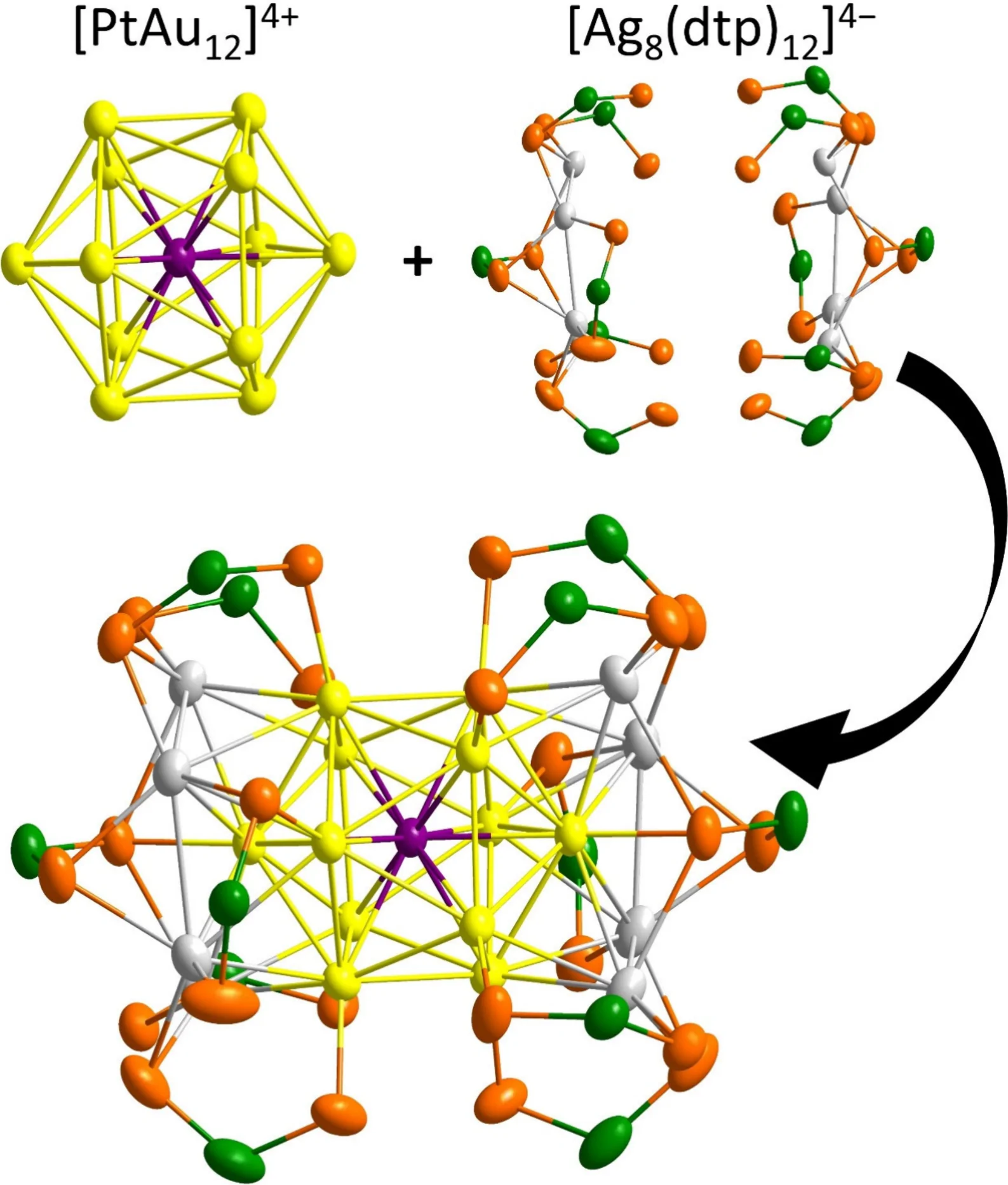
Structural analysis of **PtAu**_**12**_ **Ag**_**8**_. Overall structure of **PtAu**_**12**_ **Ag**_**8**_ and its structural decomposition into the [PtAu_12_]^4+^ icosahedral core and the [Ag_8_(dtp)_12_]^4–^ passivating layer. Color code: purple for Pt; yellow for Au; silver for Ag; orange for S; green for P.

**Table 1:** Symmetries, Icosahedral Volumes (Å^3^), Selected Bond Distances (Å), and HOMO–LUMO Gaps (eV) of **PtAu**_**12**_ **Ag**_**8**_ and **PtAg**_**20**_

	**PtAu_12_Ag_8_**	**PtAg_20_**	
symmetry	*C*_2_	*C*_1_ ^12^	*C*_3_ ^23,43^
icosahedral volume	52.26	53.87	52.76
Pt–M_ico_	2.6910(6)–2.7671(7), avg. 2.7426(7)	2.7034(7)–2.8182(7), avg. 2.7601(7)	2.7519(14)–2.7461(14), avg. 2.7505(13)
M_ico_–M_ico_	2.8109(7)–2.9617(8), avg. 2.8839(8)	2.8314(9)–3.0135(9), avg. 2.9022(9)	2.8349(16)–2.9556(16), avg. 2.9013(16)
M_ico_–Ag_cap_	2.820(1)–3.317(1), avg. 2.972(1)	2.8601(10)–3.1070(9), avg. 2.9785(9)	2.9130(18)–3.0115(16), avg. 2.9587(17)
Δ*E*_H–L_	1.25	1.56	1.91

DFT calculations on **PtAu**_**12**_ **Ag**_**8**_ were performed at the BP86/def2-TZVP level of theory (see Computational Details in the Supporting Information). To reduce computational cost, simplified models were employed, in which the O^*n*^Pr groups of the dtp ligands were replaced by hydrogen atoms. This simplification has been validated in numerous previous studies on related coinage-metal nanoclusters.^12,20,24–26,30,40,43^ Selected computed data are summarized in Table S1. The optimized geometry of **PtAu**_**12**_ **Ag**_**8**_ is in good agreement with its experimental counterpart determined by single-crystal X-ray diffraction, confirming the reliability of the simplified models for describing their electronic structures. In **PtAu**_**12**_ **Ag**_**8**_, all eight Ag atoms are located in the outer passivating layer and are each coordinated by three S atoms from dtp ligands, consistent with typical Ag­(I)-type coordination environments. Together with the 12 dtp ligands, the eight Ag atoms can be divided into two [Ag_4_(dtp)_6_]^2–^ units that encapsulate the 8-electron [PtAu_12_]^4+^ core. The Kohn–Sham orbital diagram of **PtAu**_**12**_ **Ag**_**8**_ (Figure 3a) illustrates its 1S^2^1P^6^1D^0^ superatomic configuration. The HOMO and HOMO–1 exhibit clear nodal patterns and spatial distributions characteristic of superatomic 1P orbitals. The third 1P-like component is less apparent, likely because its orientation toward the two [Ag_4_(dtp)_6_]^2–^ units leads to stronger mixing and delocalization over the passivating layer (Figure 3b). The LUMOs can be assigned to the superatomic 1D level, while NAO charges close to +0.6 for the eight peripheral Ag atoms, further corroborate their assignment as Ag­(I)-type centers in the passivating layer. The TD-DFT simulated UV–vis spectrum of **PtAu**_**12**_ **Ag**_**8**_ shows three main absorption features at approximately 360, 460, and 620 nm, which show reasonable agreement with the experimentally observed features at around 420, 520, and 700 nm (Figure 1c). While the relative energy spacings between the bands correlate well, the systematic blue shift in the calculated transitions is attributed to the simplification of the propoxy chains to hydrogen atoms in the computational model. The calculated absorptions at 460 and 620 nm mainly arise from superatomic 1P to 1D transitions. The absorption at 360 nm contains contributions from both 1P to 1D transitions and partial charge transfer from the passivating layer to the 1D level. The transformation of the eight-electron superatomic core from [PtAg_12_]^4+^ to [PtAu_12_]^4+^ leads to a reduced frontier orbital energy gap, as reflected in the TD-DFT simulated absorption spectra. As a result, the lowest-energy absorption feature of **PtAu**_**12**_ **Ag**_**8**_ is red-shifted compared with that of **PtAg**_**20**_ (Figure S4).

**Figure 3:**
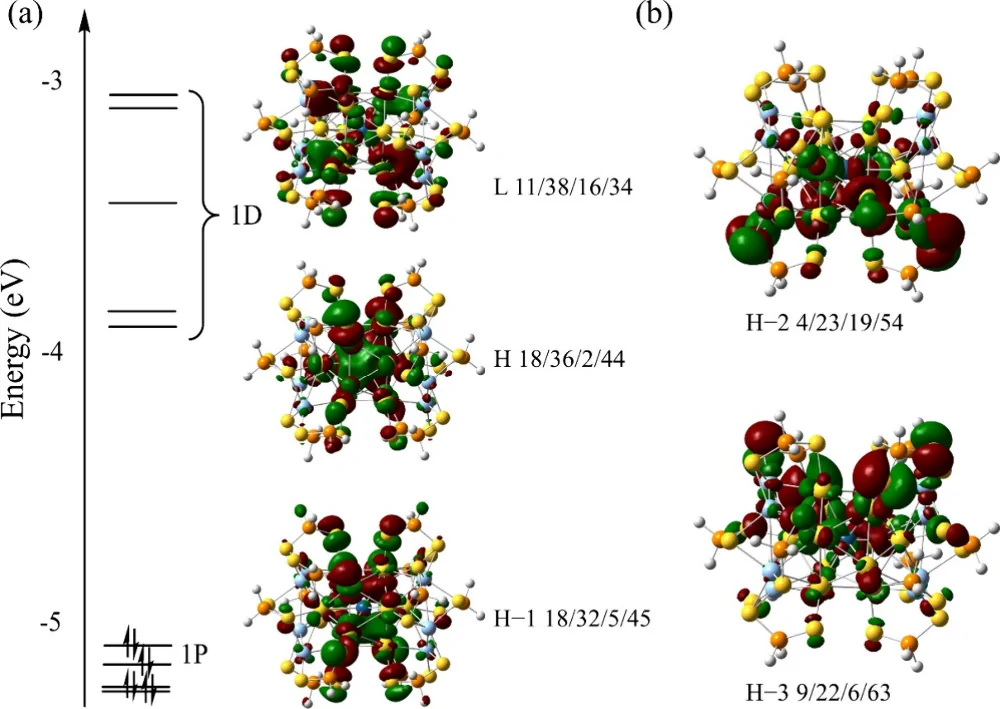
(a) Kohn–Sham molecular orbital diagram of **PtAu**_**12**_ **Ag**_**8**_. (b) The third 1P-like component affected by the passivating layer is distributed over the H–2 and H–3 orbitals. Atomic contributions (in %) are given in the order Pt/Au/Ag/ligand.

In conclusion, we have demonstrated the synthesis of **PtAu**_**12**_ **Ag**_**8**_ through redox-mediated Au-for-Ag exchange from **PtAg**_**20**_. In this transformation, the [PtAg_12_]^4+^ superatomic core is converted into a compact [PtAu_12_]^4+^ core, while the [Ag_8_(dtp)_12_]^4–^ passivating layer is retained. Compared to conventional alloy doping strategies, this methodology offers distinct advantages: it achieves complete, regioselective substitution of the 12 icosahedral cage positions without disorder or partial occupancies in the solid state. Despite the identical shell nuclearity, **PtAu**_**12**_ **Ag**_**8**_ exhibits a previously unobserved *C*_2_ passivation-layer arrangement, clearly distinct from the *C*_3_-type shells found in related [Ag_8_(dtp)_12_]^4–^-protected superatoms. DFT calculations reveal that the [PtAu_12_]^4+^ core maintains an eight-electron superatomic configuration, and TD-DFT analysis assigns the major visible absorptions mainly to 1P to 1D superatomic transitions. These findings show that site-selective replacement of the icosahedral cage metal can not only tune the electronic structure of the superatomic core but also reconstruct the outer Ag–ligand passivating layer, offering a new strategy for designing trimetallic superatoms with controlled core–shell metal distributions.

## Supplementary Material



## Notes

Experimental details, structural figures, crystallographic tables, and DFT computational results (PDF)
