# Supplementary material for: A Dichalcogenide-Protected Pt@Au12@Ag8 Nanocluster
Source: Inorg Chem. 2026 Oct 15;65(42):24640–5. doi: 10.1021/acs.inorgchem.6c04087 (PMC13715580; doi:10.1021/acs.inorgchem.6c04087)
Supplement: Supplementary file 1 [file ic6c04087_si_001.pdf]

Supplementary Information

A Dichalcogenide-Protected $Pt@Au_{12}@Ag_8$ Nanocluster

Tzu-Hao Chiu,[a] Michael N. Pillay,[a] Samia Kahlal,[b] Jean-Yves Saillard,[b]* and C. W. Liu[a]*

[a] Department of Chemistry, National Dong Hwa University, Hualien 97401, Taiwan (Republic of China)

[b] Univ Rennes, CNRS, ISCR-UMR 6226, F-35000 Rennes, France

* Correspondence: E-mail: chenwei@gms.ndhu.edu.tw; Tel: +886-3-890-3607; Fax : +886-3-890-0162; https://cwliu.weebly.com/
Keywords: superatom, trimetallic alloy, gold, silver, platinum

Experimental Section

1.1 General remarks

All chemicals were purchased from commercial sources and used as received. Solvents were purified following standard protocols. All reactions were carried out under N_2 atmosphere by using standard Schlenk techniques. These reagents are considered corrosive and should be used with caution. NMR spectra were recorded on a Bruker AVII-400 and AV-600 MHz NMR spectrometers. The chemical shift (δ) is reported in ppm and Hz, respectively. ESI-mass spectra recorded on a Bruker maXis Q-TOF mass spectrometer (Bruker Daltonik GmbH, Germany). UV–visible-NIR absorption spectra were measured on a Shimadzu UV3101PC spectrophotometer at 298 K, using quartz cells with a path length of 1 cm.

1.2 Synthesis

1.2.1 Synthesis of $PtAu_{12}Ag_8[S_2P(OPr)_2]_{12}$

$PtAg_{20}[S_2P(OPr)_2]_{12}$ (10 mg)[1,2] was placed in a Schlenk tube and dissolved in THF. $Au(SMe_2)Cl$ (20 equiv.) was then added to the solution, and the mixture was immediately evaporated to dryness under reduced pressure. The resulting residue was extracted with hexane, leaving insoluble materials behind. Evaporation of the hexane extract afforded pure $PtAu_{12}Ag_8[S_2P(OPr)_2]_{12}$.
(10 mg; Yield: 82% base on $PtAg_{20}[S_2P(OPr)_2]_{12}$)
$^{31}P\{^1H\}$ NMR (161.97 MHz, $CDCl_3$, δ, ppm, r.t.): 102.5, 101.1 ESI-MS (m/z): exp. 6087.9678 (calc. for $[M + Ag]^+$: 6087.9136) UV-vis (λ_{max} in nm, ε in M^{-1} cm^{-1}): 420 (69500), 520 (39700), 700 (15700).

1.3 X-ray crystallography

Single crystal X-ray diffraction data were collected using an XtaLAB Synergy, single source at home/near, HyPix-Arc 100 diffractometer operating at T = 100.01(10) K. Data were measured using w scans of 0.5° per frame for 0.1 s using Cu Ka radiation. The diffraction pattern was indexed, and the total number of runs and images was determined based on the strategy calculation in the CrysAlisPro program (Rigaku, Version 1.171.44.120a, 2025). Data reduction, scaling, and absorption corrections were performed using CrysAlisPro (Rigaku, V1.171.44.120a, 2025). The structure was solved with the ShelXT (Sheldrick, 2015) structure solution program using the Intrinsic Phasing solution method and by using Olex2 as the graphical interface.[3,4] Further details provided in Table S2. CCDC 2578230 contain the supplementary crystallographic data for compound **$PtAu_{12}Ag_8$** in this article. These data can be obtained free of charge from the Cambridge Crystallographic Data Centre via www. ccdc.cam.ac.uk/data_request/cif.

1.4 Computational Details

Geometry optimizations were performed by density functional theory (DFT) calculations with the Gaussian 16 package,[5] using the BP86 functional[6,7] and the all-electron Def2-TZVP set from EMSL Basis Set Exchange Library.[8,9] All the optimized geometries were characterized as true minima by vibrational analysis. The NAO charges and Wiberg bond indices were computed with the NBO 6.0 program [10,11] on single-point calculations performed with the BP86 functional and the Def2-SVP basis set for computational limitations.[12,13] The UV-visible transitions were calculated by means of time-dependent DFT (TD-DFT) calculations, with the CAM-B3LYP functional[14] and the Def2-TZVP basis set. Only singlet-singlet, *i.e.* spin-allowed, transitions have been computed. The UV-visible spectra were simulated from the computed TD-DFT transitions and their oscillator strengths by using the Multiwfn program,[15] each transition is associated with a Gaussian function of half-height width equal to 2000 cm^{-1}. The compositions of the molecular orbitals were calculated using the AOMix program.[16]

Reference

1. Chiu, T.-H.; Liao, J.-H.; Lin, Y.-R.; Liu, C. W. Surface-Induced Isomerization in [PtAg20{S2P(OR)2}12] (R = nPr, iPr, iBu) Nanoclusters: Unveiling Shell Variability through X-ray Diffraction. *Cryst. Growth Des.* **2024**, *24*, 5171–5178.
2. Chiu, T.-H.; Liao, J.-H.; Gam, F.; Chantrenne, I.; Kahlal, S.; Saillard, J.-Y.; Liu, C. W. Homoleptic Platinum/Silver Superatoms Protected by Dithiolates: Linear Assemblies of Two and Three Centered Icosahedra Isolobal to Ne2 and I3−. *J. Am. Chem. Soc.* **2019**, *141*, 12957–12961.
3. O. V. Dolomanov; L. J. Bourhis; R. J. Gildea; J. A. K. Howard; H. Puschmann. OLEX2: A Complete Structure Solution, Refinement and Analysis Program,. *J. Appl. Crystallogr.* 2009, *42*, 339–341.
4. L. J. Bourhis; O. V. Dolomanov; R. J. Gildea; J. A. K. Howard; H. Puschmann. The Anatomy of a Comprehensive Constrained, Restrained Refinement Program for the Modern Computing Environment—Olex2 Dissected,. *Acta Crystallogr. Sect. A* 2015, *71*, 59–75.
5. M. J. Frisch, G. W. Trucks, H. B. Schlegel, G. E. Scuseria, M. A. Robb, J. R. Cheeseman, G. Scalmani, V. Barone, G. A. Petersson, H. Nakatsuji, X. Li, M. Caricato, A. V. Marenich, J. Bloino, G. Janesko, R. Gomperts, B. Mennucci, H. P. Hratchian, J. V. Ortiz, A. F. Izmaylov, J. L. Sonnenberg, D. Williams-Young, F. Ding, F. Lipparini, F. Egidi, J. Goings, B. Peng, A. Petrone, T. Henderson, D. Ranasinghe, V. G. Zakrzewski, J. Gao, N. Rega, G. Zheng, W. Liang, M. Hada, M. Ehara, K. Toyota, R. Fukuda, J. Hasegawa, M. Ishida, T. Nakajima, Y.

Honda, O. Kitao, H. Nakai, T. Vreven, K. Throssell Jr., J. A. Montgomery, J. E. Peralta, F. Ogliaro, M. J. Bearpark, J. J. Heyd, E. N. Brothers, K. N. Kudin, V. N. Staroverov, T. A. Keith, R. Kobayashi, J. Normand, K. Raghavachari, A. P. Rendell, J. C. Burant, S. S. Iyengar, J. Tomasi, M. Cossi, J. M. Millam, M. Klene, C. Adamo, R. Cammi, W. J. Ochterski, R. L. Martin, K. Morokuma, O. Farkas, J. B. Foresman, D. J. Fox, Gaussian 16, Revision A.03, Gaussian, Inc., Wallingford, CT, 2016.

6. A. D. Becke. Density-Functional Exchange-Energy Approximation with Correct Asymptotic Behavior,. *Phys. Rev. A* 1988, *38*, 3098–3100.
7. J. P. Perdew. Density-Functional Approximation for the Correlation Energy of the Inhomogeneous Electron Gas,. *Phys. Rev. B* 1986, *33*, 8822–8824.
8. A. Schäfer; H. Horn; R. Ahlrichs. Fully Optimized Contracted Gaussian Basis Sets for Atoms Li to Kr,. *J. Chem. Phys.* 1992, *97*, 2571–2577.
9. A. Schäfer; C. Huber; R. Ahlrichs. Fully Optimized Contracted Gaussian Basis Sets of Triple Zeta Valence Quality for Atoms Li to Kr,. *J. Chem. Phys.* 1994, *100*, 5829–5835.
10. E. D. Glendening; C. R. Landis; F. Weinhold. Natural Bond Orbital Methods,. *J. Comput. Chem.* 2013, *34*, 1429–1437.
11. E. D. Glendening, J. K. Badenhoop, A. E. Reed, J. E. Carpenter, J. A. Bohmann, C. M. Morales, C. R. Landis, F. Weinhold, NBO 6.0, Theoretical Chemistry Institute, University of Wisconsin, Madison, WI, 2013.
12. F. Weigend; R. Ahlrichs. Balanced Basis Sets of Split Valence, Triple Zeta Valence and Quadruple Zeta Valence Quality for H to Rn: Design and Assessment of Accuracy,. *Phys. Chem. Chem. Phys.* 2005, *7*, 3297–3305.
13. F. Weigend. Accurate Coulomb-Fitting Basis Sets for H to Rn,. *Phys. Chem. Chem. Phys.* 2006, *8*, 1057–1065.
14. T. Yanai; D. P. Tew; N. C. Handy. A New Hybrid Exchange–Correlation Functional Using the Coulomb-Attenuating Method (CAM-B3LYP),. *Chem. Phys. Lett.* 2004, *393*, 51–57.
15. T. Lu; F. Chen. Multiwfn: A Multifunctional Wavefunction Analyzer,. *J. Comput. Chem.* 2012, *33*, 580–592. http://www.sg-chem.net
16. S. I. Gorelsky, AOMix program, http://www.sg-chem.net

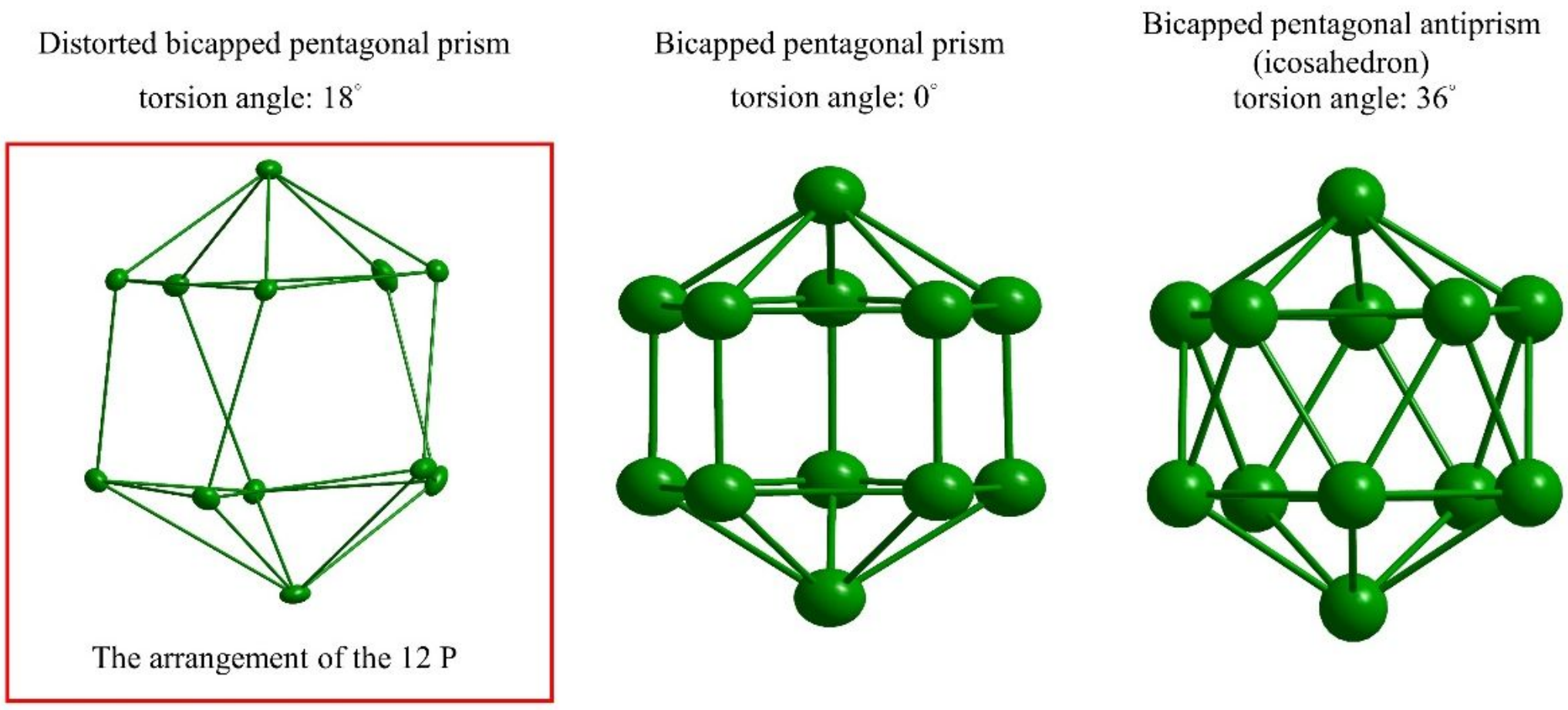

Figure S1. The arrangement of the 12 P.

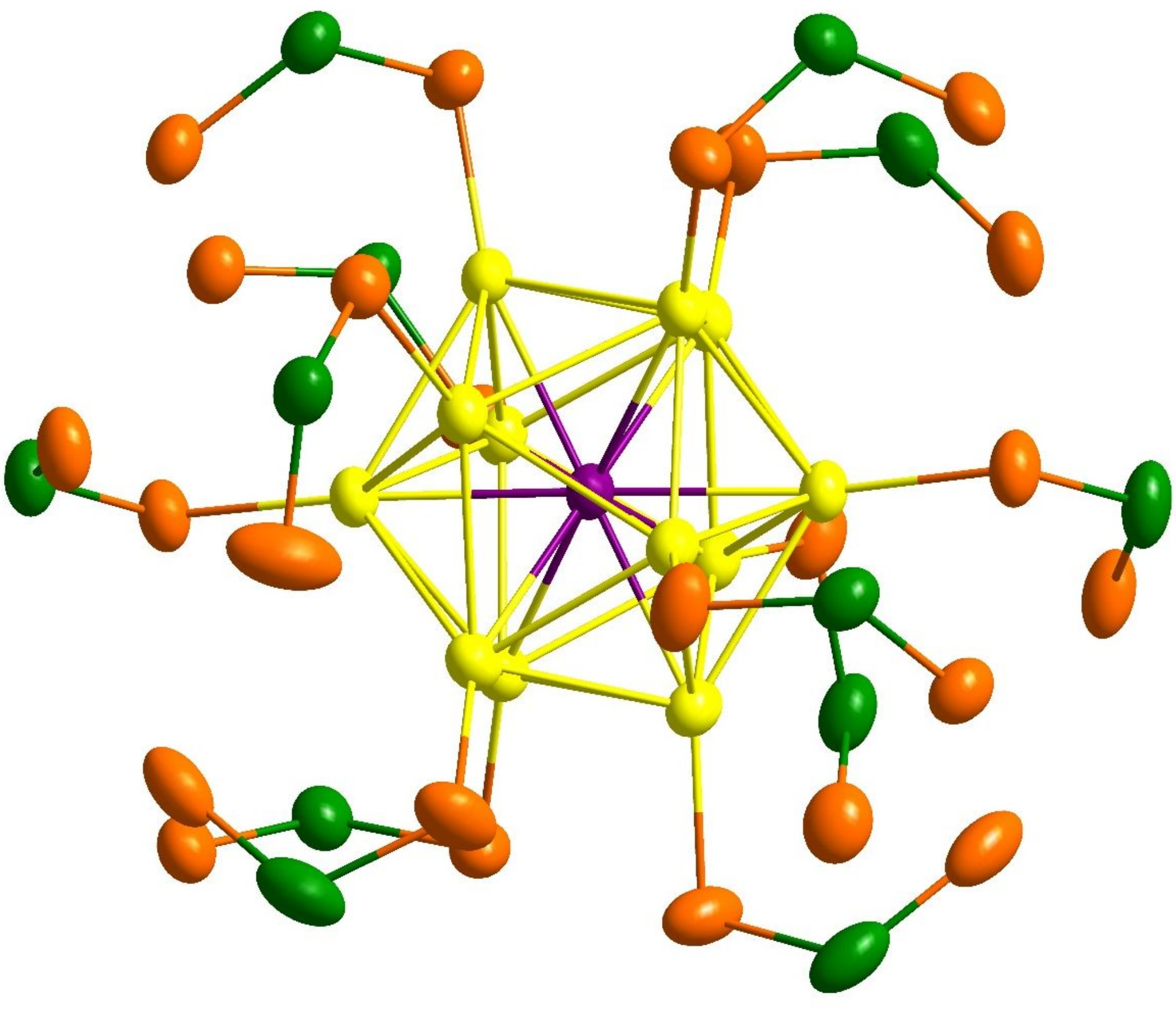

Figure S2. Structure showing the connections between the $PtAu_{12}$ core and the twelve dtp ligands. The eight Ag atoms are omitted for clarity to highlight that each Au atom is connected to only one S atom.

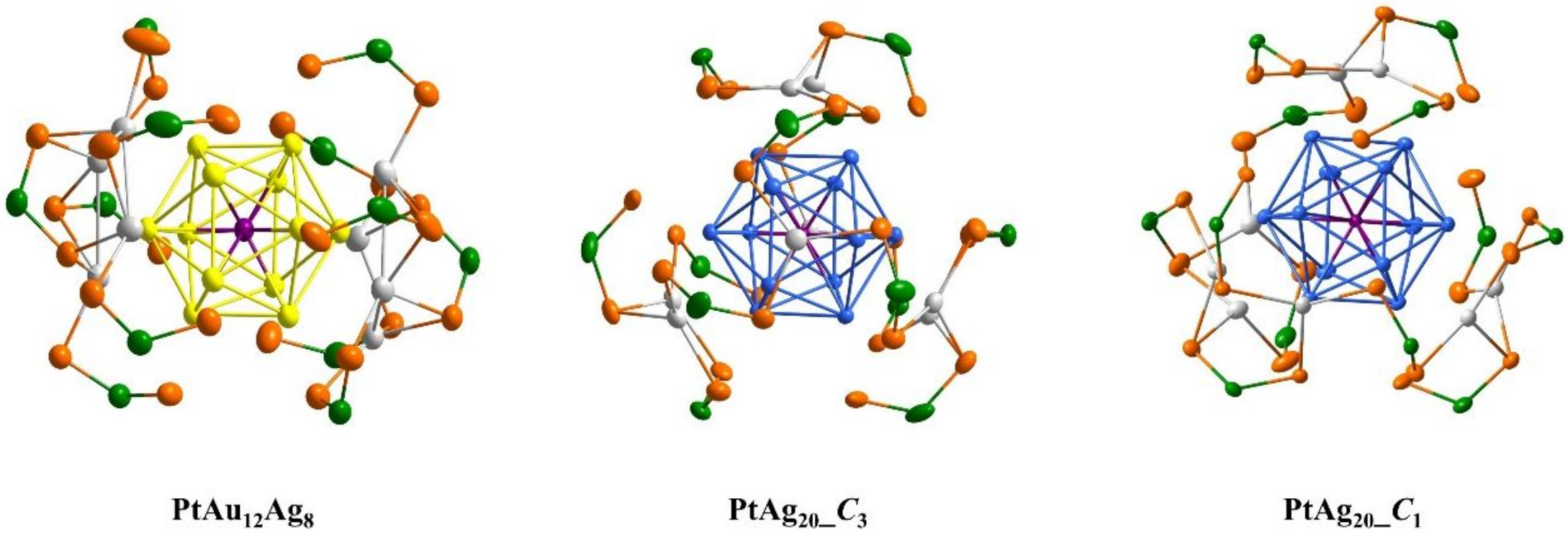

Figure S3. Comparison of the $[Ag_8(dtp)_{12}]$ passivating-layer arrangements in **$PtAu_{12}Ag_8$** and **$PtAg_{20}$.**

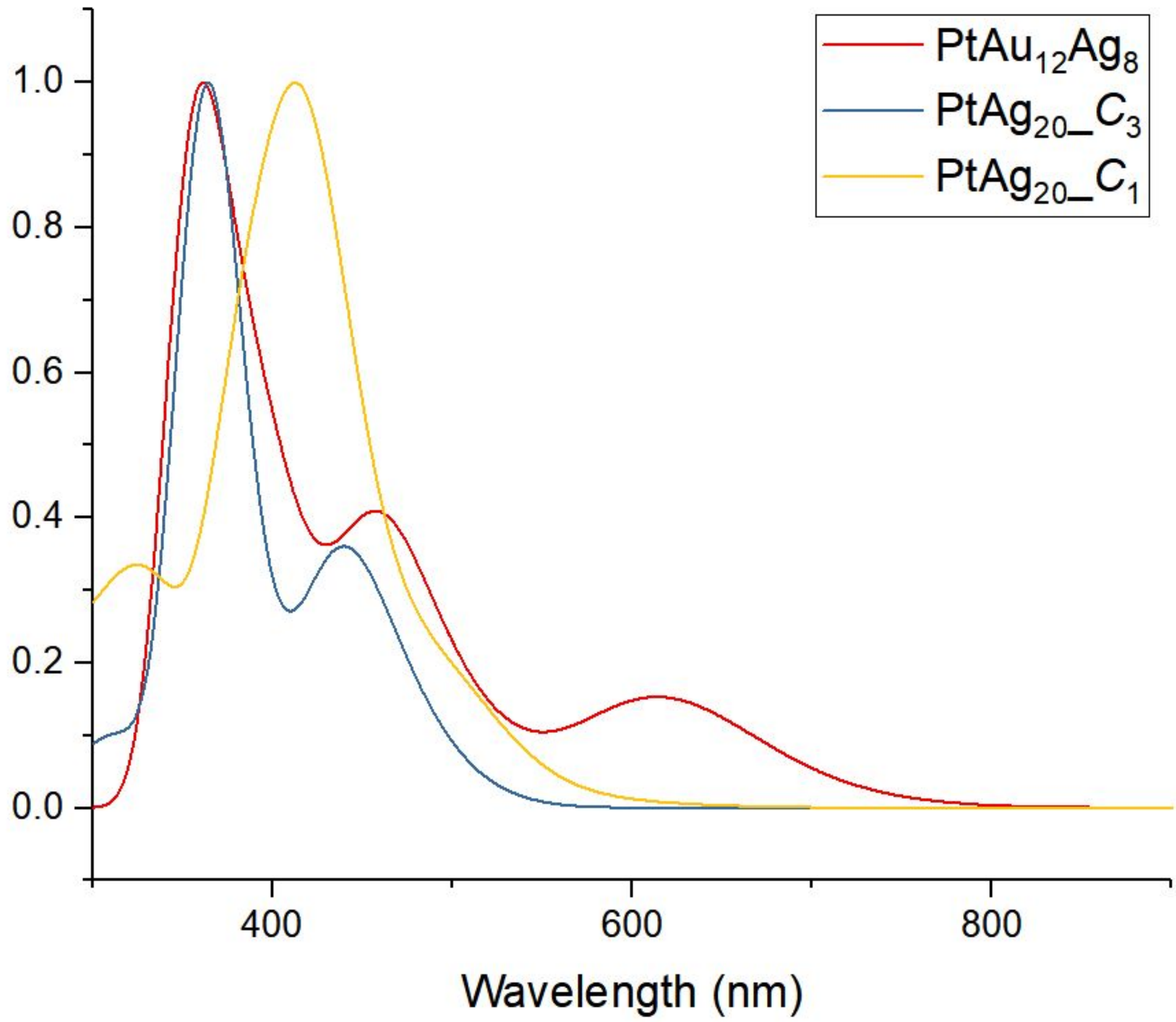

Figure S4. TD-DFT-simulated UV-vis absorption spectra of **$PtAu_{12}Ag_8$** and **$PtAg_{20}$**

Table S1. Selected Computed Distances (Å), Wiberg Bond Indices (WBIs, in brackets) and Averaged Atomic Charges for **$PtAu_{12}Ag_8$**

Cluster		**$PtAu_{12}Ag_8$**
$\Delta E_{HOMO–LUMO}$ (eV)		1.25
Pt-Au (avg.)		2.775–2.861 Å (avg. 2.821 Å) [0.125–0.148, avg. 0.138]
Au-Au (avg.)		2.869–3.065 Å (avg. 2.966 Å) [0.056–0.096, avg. 0.068]
Au-Ag (avg.)		2.862–3.158 Å (avg. 3.001 Å) [0.068–0.159, avg. 0.048]
NPA atomic charges	Pt	−0.95
	Au	0.14
	Ag	0.62

Table S2. Selected X-ray crystallographic data of $\mathbf{PtAu_{12}Ag_8}$

Compound	$\mathbf{PtAu_{12}Ag_8}$
CCDC no.	2578230
Chemical formula	$C_{72}H_{168}Ag_8Au_{12}O_{24}P_{12}PtS_{24}$
Formula weight	5980.78
Wavelength, Å	1.54184
Crystal System	Monoclinic
Space group	$P2_1/n$
a, Å	18.87580(10)
b, Å	27.8860(2)
c, Å	27.3940(2)
α, deg.	90
β, deg.	90.6140(10)
γ, deg.	90
V, $Å^3$	14418.57(17)
Z	4
Temperature, K	100(2)
ρ_{calcd}, g/cm^3	2.755
μ, mm^{-1}	37.494
θ_{max}, deg.	139.996
Completeness, %	96.8
Reflection collected / unique	76394 / 26250<br>[R(int) = 0.0611]
Restraints / parameters	968 / 1397
$^a R1$, $^b wR2$ [I > 2σ(I)]	0.0680, 0.1834
$^a R1$, $^b wR2$ (all data)	0.0734, 0.1895
GOF	1.021
Largest diff. peak and hole, e/$Å^3$	3.54 and -3.29

$^a R1 = \Sigma \mid \mid F_o \mid - \mid Fc \mid \mid / \Sigma \mid F_o \mid$. $^b wR2 = \{\Sigma[w(F_o^2 - F_c^2)^2]/\Sigma[w(F_o^2)^2]\}^{1/2}$.

Table S3. Cartesian coordinates of the DFT-optimized structure of **$PtAu_{12}Ag_8$**.

Au	0.595000	-1.468000	2.375000
Au	-1.832000	0.060000	2.105000
Au	-1.374000	-2.449000	0.408000
Au	1.377000	-2.447000	-0.407000
Au	2.663000	0.002000	0.779000
Au	0.704000	1.500000	2.296000
Au	-1.374000	2.436000	0.442000
Au	-2.663000	-0.004000	-0.779000
Au	-0.591000	-1.471000	-2.375000
Au	1.833000	0.064000	-2.105000
Au	1.366000	2.440000	-0.444000
Au	-0.707000	1.497000	-2.298000
Pt	-0.000000	-0.034000	-0.000000
Ag	3.308000	-2.576000	1.841000
Ag	4.119000	-1.830000	-1.223000
Ag	4.357000	1.356000	-1.517000
Ag	3.838000	2.798000	1.191000
Ag	-3.302000	-2.584000	-1.842000
Ag	-4.115000	-1.835000	1.227000
Ag	-4.351000	1.356000	1.517000
Ag	-3.848000	2.793000	-1.193000
S	1.811000	-4.699000	-1.205000
S	4.898000	-3.913000	0.263000
S	3.941000	-3.222000	4.164000
S	0.496000	-2.579000	4.500000
S	-4.030000	-2.587000	3.685000
S	-3.291000	0.858000	3.897000
S	-1.805000	-4.702000	1.208000
S	-4.892000	-3.916000	-0.262000
S	6.291000	-0.312000	-0.932000
S	4.669000	0.467000	2.136000
S	3.801000	4.524000	3.000000
S	1.046000	2.701000	4.315000
S	-4.671000	0.460000	-2.133000
S	-6.287000	-0.316000	0.938000
S	-0.489000	-2.583000	-4.499000

S	-3.934000	-3.228000	-4.166000
S	-1.053000	2.698000	-4.316000
S	-3.811000	4.517000	-3.002000
S	4.037000	-2.583000	-3.681000
S	3.293000	0.861000	-3.895000
S	5.157000	3.701000	-0.989000
S	1.813000	4.781000	-0.820000
S	-1.821000	4.779000	0.814000
S	-5.164000	3.697000	0.989000
P	3.429000	-5.262000	-0.135000
P	2.420000	-2.307000	5.111000
P	-4.319000	-0.825000	4.552000
P	-3.424000	-5.266000	0.139000
P	6.320000	-0.149000	1.076000
P	1.881000	4.420000	3.577000
P	-6.319000	-0.162000	-1.070000
P	-2.412000	-2.313000	-5.112000
P	-1.891000	4.416000	-3.577000
P	4.318000	-0.823000	-4.553000
P	3.598000	4.744000	-1.776000
P	-3.606000	4.745000	1.770000
H	5.684000	-0.427000	-4.602000
H	4.013000	-0.897000	-5.938000
H	4.011000	-6.363000	-0.821000
H	3.051000	-5.870000	1.093000
H	7.374000	0.715000	1.476000
H	6.734000	-1.387000	1.637000
H	3.433000	4.349000	-3.131000
H	4.003000	6.098000	-1.917000
H	1.017000	4.925000	2.568000
H	1.701000	5.371000	4.617000
H	-4.012000	6.100000	1.904000
H	-3.440000	4.358000	3.127000
H	-5.686000	-0.432000	4.590000
H	-4.024000	-0.895000	5.940000
H	-7.377000	0.695000	-1.473000
H	-6.726000	-1.404000	-1.627000
H	-1.710000	5.367000	-4.617000

H	-1.027000	4.920000	-2.567000
H	-2.665000	-0.920000	-5.231000
H	-2.427000	-2.719000	-6.474000
H	-3.046000	-5.877000	-1.087000
H	-4.006000	-6.364000	0.828000
H	2.436000	-2.713000	6.473000
H	2.672000	-0.914000	5.229000

Table S4. High-Resolution ESI-MS Spectrum and Peak Assignments for the Stepwise Au-for-Ag Transmetalation Series of $PtAu_{12}Ag_8$

Peak	Exp. m/z	Calc. m/z	Assigned Formulae
**1**	5737.7500	5737.7122	$[PtAu_8Ag_{12}\{S_2P(OPr)_2\}_{12} + Ag]^+$
**2**	5825.8096	5825.7734	$[PtAu_9Ag_{11}\{S_2P(OPr)_2\}_{12} + Ag]^+$
**3**	5909.7998	5909.8346	$[PtAu_{10}Ag_{10}\{S_2P(OPr)_2\}_{12} + Ag]^+$
**4**	5999.8911	5999.8958	$[PtAu_{11}Ag_9\{S_2P(OPr)_2\}_{12} + Ag]^+$
**5**	6087.9678	6087.9136	$[PtAu_{12}Ag_8\{S_2P(OPr)_2\}_{12} + Ag]^+$
**6**	6179.9941	6179.9748	$[PtAu_{12}Ag_8\{S_2P(OPr)_2\}_{12} + Au]^+$

Note: Calculated *m/z* values represent the apex centroid of the simulated isotopic abundance envelope. Intermediate satellite envelopes spaced by ~ 44 Da originate from in-source loss of a propyl fragment ($-C_3H_7$/$-C_3H_8$, $\Delta m \approx$ 43-44 Da), from the ligand shell during electrospray ionization.

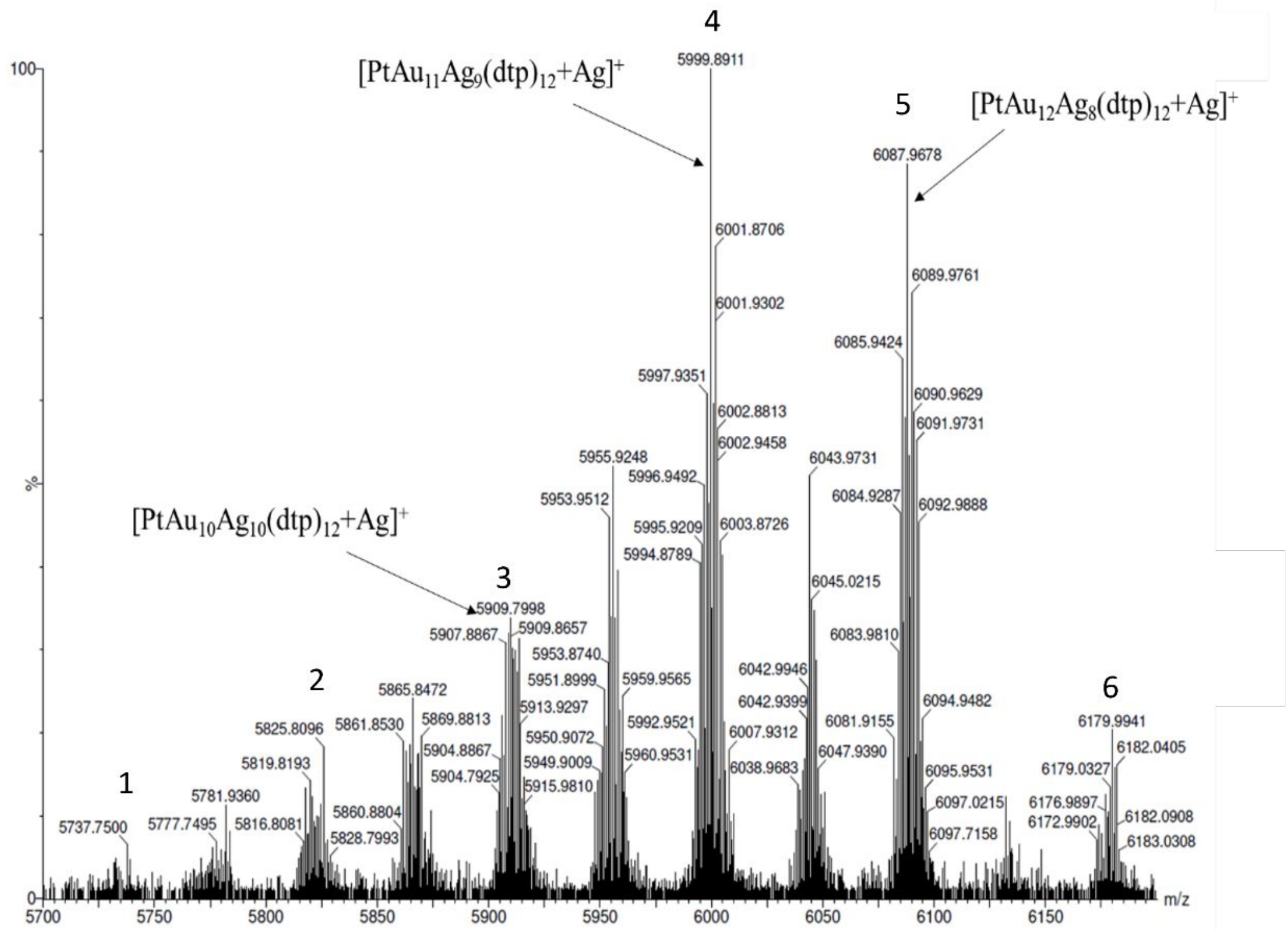

Figure S5. mode ESI-MS spectrum of $PtAu_{12}Ag_8[S_2P(OPr)_2]_{12}$ (*m/z* 5700–6200) showing the stepwise Au-for-Ag exchange manifold and competitive $[M+Ag]^+$ versus $[M+Au]^+$ adduction.
